# Cognitive impairment and dependence of patients with diabetes older than 65 years old in an urban area (DERIVA study)

**DOI:** 10.1186/s12877-016-0208-3

**Published:** 2016-02-01

**Authors:** Emiliano Rodríguez-Sánchez, Sara Mora-Simón, María C. Patino-Alonso, Diana Pérez-Arechaederra, José I. Recio-Rodríguez, Manuel A. Gómez-Marcos, Luis F. Valero-Juan, Luis García-Ortiz, for the DERIVA Group

**Affiliations:** Primary Care Research Unit, The Alamedilla Health Center, Castilla and León Health Service (SACyL), Biomedical Research Institute of Salamanca (IBSAL), Salamanca, Spain; Medicine Department, University of Salamanca, Salamanca, Spain; Basic Psychology, Psychobiology and Behavioral Sciences Methodology Department, University of Salamanca, Salamanca, Spain; Statistics Department, University of Salamanca, Salamanca, Spain; Preventive Medicine, Public Health and Medical Microbiology Department, University of Salamanca, Salamanca, Spain; Biomedical and Diagnostic Sciences Department, University of Salamanca, Salamanca, Spain

**Keywords:** Aging, Cardiovascular risk, Cognitive impairment, Dementia, Dependence, Depression, Diabetes

## Abstract

**Background:**

We analyzed the associations between diabetes and cognitive impairment (CI) and dependence in a population of patients 65 years or older.

**Methods:**

Cross-sectional study. We randomly selected 311 participants over the age of 65 living in an urban area of Spain. The mean age of the cohort was 75.89 ± 7.12 years, and 69 of the individuals (22.2 %) had diabetes. Two questionnaires were used to assess cognitive performance (MMSE and Seven Minute Screen Test), and two assessments were used to evaluate patient dependence (Barthel Index and Lawton-Brody Index). Clinical information and sociodemographic data were also gathered.

**Results:**

Nearly one quarter of patients with diabetes (21.7 %) lived alone. Diabetic patients were more sedentary (*p* = .033) than non-diabetic patients. Roughly one sixth (15.3 %) of the diabetics and 10.1 % of the non-diabetics were depressed (*p* = .332). CI was present in 26.1 % of the diabetics and 14.5 % of non-diabetics (*p* = .029). Diabetic patients had a MMSE score that was significantly worse than non-diabetics (24.88 ± 4.74 vs 26.05 ± 4.03; *p* <.05), but no differences were found in the Seven Minute Screen Test. Logistic regressions revealed that the presence of diabetes was independently associated with CI (adjusted for age, gender, years of education, sedentary lifestyle, body mass index, diastolic blood pressure, cholesterol, and depression (OR = 2.940, *p* = .013). Patients with diabetes showed greater dependence, as measured by the Barthel Index (*p* = .03) and Lawton-Brody Index (*p* <.01). Nevertheless, when dependence (dependence or not dependence for each questionnaire) used as a dependent variable in the logistic regression analyses, no significant association with diabetes was found, after adjusting for confounding variables.

**Conclusions:**

Diabetic patients over the age of 65 are more likely to present CI but not dependence. These findings support the need to include both a functional and cognitive assessment as necessary components in a standard evaluation in both clinical guides and randomized trials of therapeutic interventions in patients with diabetes.

## Background

At present, more than 20 % of individuals over the age of 65 suffer from diabetes [1, 2]. Furthermore, the prevalence of cognitive impairment (CI) has reached similar levels [3, 4]. These figures are expected to continue to grow in the coming years, due to the progressive aging of the population [3]; the prevalence of dependence is also expected to increase.

A number of studies have found an association between diabetes mellitus and increased risk of CI [5–8]. However, only a few studies have used a comprehensive neuropsychological battery of tests to study CI in diabetics, and these studies have produced contradictory results [9–11]. While some studies found an increase in the presence of CI in diabetic patients [12, 13], others suggested that diabetes is associated with a slower rate of cognitive decline in Alzheimer’s disease [14]. These discrepancies can be explained by a number of factors [15–17], including differences in study method and design, demographic characteristics (i.e., subject age, level of education), presence of diabetes, the prevalence of risk factors, and geographical scope [9–12]. It is not clear whether the risk of CI is equally associated with diabetes throughout the world or whether it is linked to subgroups that are characterized by varying levels of disease severity presence of comorbidities [18, 19].

It is also possible that differences found in the association between CI and diabetes are related to the diagnostic criteria used to define CI and dementia [20]. Although the diagnostic criteria for CI and dementia take into account the degree of dependence of the subject, little research has been conducted on the differences between countries in terms of diabetes and dependence [21]. Peripheral artery disease and peripheral nerve dysfunction are known causes of diabetes -related disability, and may explain more than 30 % of the association of diabetes with disability related dysfunction in physical activity. Physical activity is particularly important for people with diabetes, because being physically active can improve the body's ability to use insulin and facilitate weight loss [21].

The purpose of this study was to analyze the association of diabetes with CI and dependence in a population sample of patients age 65 and older.

## Methods

### Study design

An observational, descriptive, cross-sectional population study.

The initial sample for this study was taken from the DERIVA (DEmentia and cardioVAscular RIsk) study, whose methodology has already been published elsewhere [4]. The purpose of the DERIVA study was to explore the needs for services that promote personal autonomy and care for people older than 65. The reference population was the population of the city of Salamanca (172,375 inhabitants total), of which 19.74 % (34,020) were aged 65 or older. The sample was taken from the Castilla and León Regional Health Service lists, which cover 99.5 % of the population. The lists included both community dwellers and institutionalized elders. This study was carried out via home interviews in the urban area of Salamanca, Spain. The study was approved by an independent ethics committee of the Salamanca University Hospital (Spain), and all participants were provided with written informed consent, according to the recommendations of the Declaration of Helsinki.

### Subjects

In the DERIVA study, a random sample was selected, and was composed of 327 subjects that represented the adult population of Salamanca age 65 and older. To obtain this random sample, we performed stratified random sampling by health districts. Exclusion criteria included: 1) deceased individuals, 2) errors in address, 3) people who had moved out of the study area, and 4) individuals who declined to participate in the study. In total, 16 participants did not have a complete cognitive assessment; therefore 311 participants were included in this study.

### Variables

#### Cognitive impairment

Participants were defined as having CI if they met the following criteria [20]: 1) performance on neuropsychological tests that was 1.5 standard deviations below published norms on any test according to age and education level), which indicated impairment in one or more cognitive domains, 2) functional impairment on instrumental or basic activities of daily living, and 3) concern about a change in cognitive or functional levels compared with his or her previous state via self-report or a reliable informant. We also included those patients taking anti-dementia medication.

We assessed cognitive performance using the Spanish version of the Mini-Mental State Examination (MMSE) [22], which was administered by a trained interviewer, and the Seven Minute Screen Test [23, 24]. The MMSE assesses a participant’s general cognitive state, and specific components of temporal and spatial orientation, attention and calculation, memory, language, and visuoconstructional skills. The total score of the exam ranges from 0–30 points. The Seven Minute Screen Test is composed of four tasks that also assess the patient’s general cognitive state and temporal orientation; specifically, it includes the Benton Temporal Orientation Test, the Free and Enhanced Cued Recall test (to assess episodic memory), the Clock Drawing test (to assess visuoconstructional skills), and the Categorical Fluency Task test (to assess language). The patient’s total score is the sum of the four scores obtained for each task [24].

#### Diabetes

Diabetes was assessed by self-report, which is considered to be a reliable and valid indicator of a diabetic status. We also assessed diabetes based on values of basal glycemia, where values exceeding 126 mg/d in at least two determinations [25].

#### Dependence

We measured this variable using two self-reported questionnaires: the Barthel Index [26] and the Lawton and Brody Index [27]. We classified the subjects as either independent (100 points) or dependent (fewer than 100 points) in basic Activities of Daily Living (ADLs), according to the amount of help they needed in the Barthel Index. The subjects were classified as dependent (fewer than 8 points) or independent (8 points) in instrumental ADLs, according to their score on the Lawton and Brody Index.

#### Depression

We identified subjects with this condition by a diagnosis of depression or with redemption of at least 1 prescription for an antidepressant during last month.

We used the Older Americans Resources and Services Multidimensional Functional Assessment Questionnaire (OARS) to assess these variables [28], and we also administered the Spanish-validated survey from the MONICA study to evaluate cardiovascular risk factors [29]. Sociodemographic data (age, gender, marital status) and years of education were recorded. We classified each patient’s level of education as illiterate (fewer than four years), primary-secondary education (four to nine years of education), and higher education (nine years). The participants were asked whether they lived alone or were accompanied, if they were with or without a partner, and what person would be able to help them if they needed support for ADLs.

#### Clinical information

Weight, height, body mass index (BMI), waist circumference (cm), systolic blood pressure, and diastolic blood pressure were measured, and pharmacological treatments taken in the last month were noted. We determined blood sugar values and cholesterol levels from blood samples, and we calculated cardiovascular risk factors [29] and diseases diagnosed from the Charlson Comorbidity Index [30]. Cardiovascular risk factors were defined as: a) smoking: regular consumption of tobacco in the last year, and b) a sedentary lifestyle (i.e., not participating in moderate intensity physical exercise (walking 4–5 km/h) for at least half an hour, five days per week). We defined participants as suffering from hypertension, hypercholesterolemia, or cerebrovascular disease if a previous diagnosis (these documents were provided to us), if they received drug treatment for the control of these processes, or if the values recorded upon examination were higher than the following limits: a) hypertension: the average value of the second and third arm measurement was greater than 140/90, and b) hypercholesterolemia: total cholesterol ≥ 240 mg/dl.

### Statistical analyses

We checked the data for statistical normality using the Kolmogorov-Smirnov test. Normally distributed continuous variables were expressed as mean ± standard deviation; non-normally distributed variables were presented as median and 25–75^th^ percentile. We employed a frequency distribution similar in the qualitative variables. A Student’s t-test or Mann-Whitney U test was used to test the relationship between the quantitative variables. We used a chi-squared test for the qualitative variables. We conducted four logistical regression analyses to study the association of diabetes with CI, dependence (Barthel Index, Lawton and Brody Index), and depression, where CI, dependence, and depression were the dependent variables. For each dependent variable, the same variables were used to adjust the models. The first model was adjusted for age and gender. The second model was adjusted for age, gender, years of education, sedentary lifestyle, and BMI. The third model was adjusted for age, gender, years of education, sedentary lifestyle, BMI, diastolic blood pressure, and total cholesterol. The fourth model was adjusted for age, gender, years of education, sedentary lifestyle, BMI, diastolic blood pressure, total cholesterol, and depression. We analyzed the data using the Statistical Package for the Social Sciences version 20.0 statistical package (SPSS Inc., Chicago, Illinois, USA). A value of *p* <.05 was considered to be statistically significant.

## Results

The average age of the 311 participants was 75.89 ± 7.12 years, and 60.9 % of the participants were women. Diabetes mellitus was observed in 69 patients (22.2 %). When analyzing the sociodemographic characteristics of diabetics and non-diabetics, no significant differences were found between the two groups (Table 1). In general, we found that most of the participants in both groups were women, most had received Primary – Secondary education, that their partner was their main source of help for performing ADLs. Finally, nearly one quarter of patients with diabetes (21.7 %) lived alone.

**Table 1:** Socio-demographic characteristics of the participants comparing patients with diabetes and without diabetes

	Non Diabetic	Diabetic	*P* value
	*n* = 242	*n* = 69	
	Mean/Median/n	Mean/Median/n	
	SD/IQR/(%)	SD/IQR/(%)	
Age (years)	75.60 ± 7.10	76.90 ± 7.22	0.183
Male	75.18 ± 6.53	76.44 ± 7.09	
Female	75.82 ± 7.40	77.19 ± 7.38	
Age groups (years), *n*(%)			0.100
66–74	116 (47.9)	25 (36.2)	
> 75	126 (52.1)	44 (63.8)	
Gender Male, *n*(%)	84 (34.7)	27 (39.1)	0.569
Years of education, median(IQR)	9 (6–9)	9 (6–9)	0.253
Educational level, *n*(%)			0.334
Iliterate	69 (28.5)	26 (37.7)	
Primary-Secondary education	136 (56.2)	33 (47.8)	
Higher education	37 (15.3)	10 (14.5)	
Living with his/her partner, *n*(%)	139 (57.4)	40 (58.0)	1.000
Living alone, *n*(%)	46 (19.0)	15 (21.7)	0.608
Someone helps him/her with activities of daily living, *n*(%)	115 (47.5)	37 (53.6)	0.415
Who is his/her primary aid, *n*(%)			0.594
His/her partner	55 (64.7)	20 (63.5)	
Son/daughter	23 (27.1)	10 (32.3)	
Others	7 (8.2)	1 (3.2)	
Number of living children, median (IQR)	2 (1–3)	2 (1–3)	0.743

Values are means ± standard deviations(SD) for normally distributed continuous data and medians (interquartile range (IQR)) for asymmetrically distributed continuous data and number and proportions for categorical data

As can be seen in Table 2, diabetic patients were more likely to be sedentary (*p* = .033) than non-diabetic patients, had lower diastolic blood pressure (*p* = .022), and lower total cholesterol and high-density lipoprotein (HDL) levels (*p* <0.05). Roughly one sixth (15.3 %) of the diabetics and 10.1 % of the non-diabetics were depressed (*p* = .332), and CI was present in 26.1 % of the diabetics and 14.5 % of non-diabetics (*p* = .029).

**Table 2:** Cardiovascular risk factors and comorbidity of the participants comparing patients with diabetes and without diabetes

	Non Diabetic	Diabetic	*P* value
	*n* = 242	*n* = 69	
Clinical variables	Mean/Median/n	Mean/Median/n	
	SD/IQR/(%)	SD/IQR/(%)	
Smoking, *n* (%)	19 (7.9)	5 (7.2)	1.000
Sedentary lifestyle, *n* (%)	61 (25.2)	27 (39.1)	0.033
Body mass index, (kg/m2)	27.5 ± 4.4	28.2 ± 4.9	0.255
Waist circumference (cm)	100 (91–108)	101 (94–110)	0.293
Systolic blood pressure (mmHg)	138 ± 20	138 ± 22	0.786
Diastolic blood pressure (mmHg)	80 ± 13	76 ± 11	0.022
Mean blood pressure (mmHg)	99 ± 13	97 ± 13	0.185
Total cholesterol (mg/dL)	207 ± 34	190 ± 37	0.002
LDL-cholesterol (mg/dL)	124 ± 29	115 ± 30	0.053
HDL-cholesterol (mg/dL)	60 ± 17	53 ± 14	0.005
Serum glucose (mg/dL)	94 (87–102)	121 (102–146)	<0.01
Hypertension, *n* (%)	168 (69.4)	48 (69.6)	1.000
Hypercholesterolemia, *n* (%)	59 (24.4)	19 (27.5)	0.637
Depression/anxiety, *n* (%)	37 (15.3)	7 (10.1)	0.332
Cognitive impairment, *n* (%)	35 (14.5)	18 (26.1)	0.029
Cerebrovascular disease, *n* (%)	7 (2.9)	6 (8.7)	0.044
Charlson morbidity index adjusted for age	4 (3–5)	5 (4–6)	<0.01

Values are means ± standard deviations (SD) for normally distributed continuous data and number, medians (interquartile range (IQR)) for asymmetrically distributed continuous data and proportions for categorical data

*LDL* low density lipoprotein, *HDL* high density lipoprotein

According to cognitive performance assessments, patients with diabetes had poorer total scores (24.88 ± 4.74) than patients without diabetes (26.05 ± 4.03) (*p* <0.05) on the MMSE, but there were no differences in the Seven Minute Screen Test (Table 3). However, these differences were not seen when the cognitive components were analyzed separately.

**Table 3:** Cognitive performance of the participants comparing patients with diabetes and without diabetes

Neuropsychological variables	Non Diabetic	Diabetic	*P* value
	*n* = 242	*n* = 69	
	Mean/SD	Mean/SD	
Mini-mental-state examination			
Total	26.05 ± 4.03	24.88 ± 4.74	0.044
Temporal orientation	4.43 ± 1.09	4.13 ± 1.40	0.064
Spatial orientation	4.85 ± 0.57	4.87 ± 0.57	0.814
Attention and calculation	4.12 ± 1.45	3.74 ± 1.85	0.076
Memory	4.70 ± 1.20	4.39 ± 1.19	0.061
Language	7.41 ± 1.03	7.28 ± 1.16	0.355
Visuoconstructional skills	0.55 ± 0.49	0.48 ± 0.50	0.325
7 minute screen test			
Total	139.34 ± 26.20	138.68 ± 24.14	0.859
Benton temporal orientation test	103.75 ± 22.23	98.90 ± 27.55	0.136
Enhanced Cued Recall test	14.70 ± 2.48	15.11 ± 1.23	0.201
Clock drawing test	5.51 ± 2.00	5.30 ± 2.36	0.469
Category fluency	14.46 ± 6.20	14.26 ± 5.25	0.816

Values are means ± standard deviations (SD) for normally distributed continuous data

In terms of dependence (Table 4), patients with diabetes exhibited greater dependence in performing basic ADLs as measured with the Barthel Index (*p* = .03), and in performing instrumental ADLs as measured with the Lawton and Brody Index (*p* <.01). Diabetic patients showed more dependence both in their total score (*p* = .005) and in five out of the eight activities that we evaluated.

**Table 4:** Functional disability assessment of the participants comparing patients with diabetes and without diabetes

Level of dependence/ independence of ADL	Non Diabetic	Diabetic	*P* value
	*n* = 242	*n* = 69	
	Mean/n	Mean/n	
	SD/(%)	SD/(%)	
Basic activities (Barthel Index)			
Global mean	91.93 ± 18.09	88.62 ± 19.64	0.190
Independence, *n* (%)	183 (75.6)	43 (62.3)	0.033
Instrumental activities (Lawton and Brody Index):			
Global mean	6.91 ± 1.94	6.12 ± 2.47	0.005
Independence to perform eight ADL *n* (%)	153 (64.3)	35 (50.7)	0.050
Independence to perform instrumental ADL *n* (%):			
Ability to handle finances	211 (87.6)	55 (79.7)	0.117
Shopping	185 (77.1)	48 (69.6)	0.208
Responsibility for own medications,	216 (90.0)	55 (79.7)	0.035
Food preparation	183 (75.9)	43 (62.3)	0.031
Ability to use telephone	232 (96.7)	64 (92.8)	0.174
Housekeeping	208 (86.3)	49 (71.0)	0.006
Mode of transportation	233 (96.7)	62 (89.9)	0.049
Laundry	194 (80.5)	46 (66.7)	0.022

Values are means ± standard deviations (SD) for normally distributed continuous data and proportions for categorical data

*ADL* activities of daily living

Table 5 lists the results of the four logistic regressions. In the first analysis, we used CI as a dependent variable in all of the models. We found that diabetes was significantly associated with CI, even when adjusting for all covariates, showing an OR of 2.940 (IC 95 % 1.259–6.866, *p* = .013). Nevertheless, after adjusting for potential confounding variables including dependence (as assessed with the Barthel Index and the Lawton and Brody Index), the association was no longer significant. No association between diabetes and depression was found either.

**Table 5:** Multiple logistic regression analyses considering cognitive impairment, dependence by Barthel Index and Lawton and Brody Index, and depression as dependent variables, and diabetes as independent variable

Cognitive impairment			
	OR	IC 95 % OR	*P* value
Model 1	2.036	1.044–3.970	0.037
Model 2	2.384	1.142–4.979	0.021
Model 3	2.746	1.213–6.217	0.015
Model 4	2.940	1.259–6.866	0.013
Dependent variable: Cognitive Impairment. Independent variable: Diabetes			
Dependence by Barthel Index			
	OR	IC 95 % OR	*P* value
Model 1	1.794	0.951–3.386	0.071
Model 2	1.514	0.771–2.974	0.228
Model 3	1.383	0.641–2.987	0.409
Model 4	1.398	0.646–3.023	0.394
Dependent variable: Dependence by Barthel Index. Independent variable: Diabetes			
Dependence by Lawton and Brody Index			
	OR	IC 95 % OR	*P* value
Model 1	0.630	0.355–1.118	0.114
Model 2	0.635	0.351–1.151	0.134
Model 3	0.539	0.274–1.060	0.073
Model 4	0.517	0.258–1.034	0.062
Dependent variable: Dependence by Lawton and Brody Index. Independent variable: Diabetes			
Dependence by depression			
	OR	IC 95 % OR	*P* value
Model 1	0.881	0.350–2.220	0.789
Model 2	0.876	0.343–2.234	0.781
Model 3	0.892	0.344–2.315	0.814
Dependent variable: Depression. Independent variable: Diabetes			

All models were adjusted for: Model 1: Age and Gender. Model 2: Age, Gender, Years of education, sedentary lifestyle, and BMI. Model 3: Age, Gender, Years of education, sedentary lifestyle, BMI, Blood Pressure Diastolic, and Cholesterol total. Model 4: Age, Gender, Years of education, sedentary lifestyle, BMI, Blood Pressure Diastolic, Total cholesterol and depression

## Discussion

Our results show that diabetic patients over the age of 65 are more than twice as likely to show a risk association with CI and that the presence of dependence does not contribute to this association.

There are an increasing number of studies that suggest that diabetes is a risk factor for dementia, although we do not know much about the factors that contribute to this association [16, 17]. Our conclusions were drawn from a randomly selected sample of individuals living in an urban Spanish locality and are consistent with those found in a meta-analysis conducted by Cheng et al. [31]. According to those authors, people with diabetes are between 1.5 and 2.9 times more likely to be at risk for CI. Our investigation is the first study to describe the association between diabetes and CI in Spanish patients older than 65 years of age. Similar results were found in recent studies of various populations [13], including Mexicans [10], Mexican-Americans [12], Chinese [9], and Australians [16]. However, it should be noted that prospective studies such as the Framingham Heart Study [32] and the Leiden 85-plus study [33] have found an ambiguous relationship between diabetes and dementia; even a multi-center study in France found that CI progressed more slowly in groups of diabetic patients than in non-diabetic patients [14].

We measured poorer MMSE scores in patients with diabetes, which was also found by Umegaki et al. [34]. This result is consistent with the findings of Okereke et al. [35], who observed that cognitive scores were significantly lower in diabetics with a longer duration of the disease in a community-dwelling older adults population from the Physicians’ Health Study II and the Women’s Health Study. Also, the observed effect of having diabetes was the cognitive equivalent off adding three years to the patient’s age. Roberts et al. [36] observed an association between brain imaging (with ischemic and atrophic changes) and poorer cognitive performance in a population-based cohort that was free of dementia and whose diabetes had set in before the age of 64. This association was not observed in patients whose diabetes appeared at a later age. It is possible that selective mortality occurs when both diseases appear, which makes it difficult to determine the epidemiological factors that create associations among both processes [16, 19]. Also, it is probable that the pathophysiologic processes that enhance both processes may require decades to manifest themselves [36]. Zilkens et al. [16] suggested that dementia onset begins an average of two years earlier in diabetic patients and increases age-specific mortality rates. These studies suggested that it is important to control the cognitive state of diabetics even before they reach the age of 65. This same age was recommended by the consensus document from the International Association of Gerontology and Geriatrics, the European Diabetes Working Party for Older People [37], the American Diabetes Association and the American Society of Geriatrics [2].

However, it cannot be confirmed that the MMSE is a sufficient test for the early detection of CI in the diabetic population. There is still no consensus on the battery of tests that should be used for CI screening in patients with this pathology, since it has been only recommended the use of standardized evaluation instruments [20, 38]. While the MMSE appears to be more sensitive than the Seven Minute Screen Test, it is not clear why poorer scores were not obtained on the Seven Minute Screen total score test or in any of the cognitive areas that were evaluated by both tests. Additional studies are needed to clarify which alterations are present in patients with diabetes [13, 37, 39, 40], and to determine if they could be related to any specific type of CI. According to this line of thinking, several indices from resting-state EEG recordings have been proposed for this purpose, and they could be employed to track the cognitive functioning of diabetic patients and also to help in the diagnosis of those who develop CI [41].

Cognitive dysfunction is associated with decreased ability to conduct personal care and decreased adherence to anti-diabetic treatment plans [31, 42–45]. The association between diabetes and CI takes on special relevance in elderly patients who find themselves in vulnerable situations [40]; it is difficult for such patients to overcome the most dangerous complications. This limitation is serious if patients require help even before the occurrence of event that necessitates urgent attention; the situation is particularly dire for the 21 % of individuals living alone [46]. This solitude can have disastrous consequences for the patient [47]. We noted that on average, patients with diabetes have a greater need for assistance with ADLs than non-diabetic patients. The results of our study are the first to provide data about the current situation of elderly adults (age 65 or older) in Spain. Our results are similar to those of Gregg et al. [48] and McGuire et al. [45].

However, it should be noted that the association between diabetes and level of dependence disappears when controlling for potential confounding variables in the multivariate analysis. Therefore, our results revealed that diabetes is not associated with degree of dependence. This finding may be because dependence is not a personal characteristic but is instead a gap between one’s ability and one’s personal needs, as suggested by Verbrugge and Jette [49]. These authors highlighted the importance of carrying out environmental, social, and individual adaptations that make it possible for elders to perform their ADLs, and to lessen the functional impact of their physical or cognitive deficits. In fact, it has been observed that the considerable differences in the relationship between diabetes and ADL limitations exist among different countries [21, 31]. Furthermore, Bardenheier et al. [50] suggested that functional decline and physical disabilities contribute to an increased incidence of diabetes. Therefore, it seems advisable to carry out an assessment of an elderly diabetic patient’s ADL functionality in order to establish a successful treatment plan [51, 52].

Unfortunately there is no test that assesses the dependence as internationally accepted as the diagnosis criteria of diabetes. In fact, the recent publication of the diagnosis criteria from the DSM-5 and CIE for mental disorders is incomplete and problematic regarding to psychiatric impairment models and dependence. This problem is found to be more complex when the ability to perform activities of daily living is compared by countries and diseases. This is the reason why, in this study, we have used two of the most international widely used tests in the geriatric assessment, the Barthel Index and Lawton and Brody Index [53].

Links between diabetes, depression, and Alzheimer´s Disease have been established, although the exact nature of these links is not yet well understood. Less clear is the effect that the comorbidity between depression and diabetes has on cognitive impairment. In our study, we found no association between diabetes and depression. However, as published in another paper about the DERIVA study [4], our results support the link between comorbid diabetes and depression and risk for cognitive impairment. As far as we know, the relationship between these diseases (depression, diabetes, and cognitive impairment) has been analyzed in just one study [54], which found that concomitant depression and diabetes significantly increased the risk of presenting mild cognitive impairment, but the exact nature of the effects that comorbid diabetes and depression may have on it were not conclusive.

The main limitation of this study was that it is a cross-sectional study that included a relatively small number of participants. It was not possible to determine a causal relationship, nor can we confirm the association between CI and several diabetes-related clinical parameters, because we did not gather information about the length of diabetes diagnosis or if the patients had a history of hypoglycemia.

## Conclusions

The results of this study indicate that people over the age of 65 who have diabetes are more likely to present CI but not dependence. To confirm the existence of this possible effect and to further understand the exact nature of the relationship, longitudinal studies that include a large cohort of patients are needed to evaluate cognitive changes and dependence in diabetic patients. These findings support the need to include functional and cognitive assessments as integral components of the standard evaluation of patients with diabetes, in both clinical guides and randomized trials of therapeutic interventions.

## Abbreviations

ADL: Activities of Daily Living
CI: Cognitive impairment
DERIVA: DEmentia and cardioVAscular RIsk
MMSE: Mini Mental State Examination
